# Effects of spinetoram and glyphosate on physiological biomarkers and gut microbes in *Bombus terrestris*

**DOI:** 10.3389/fphys.2022.1054742

**Published:** 2023-01-09

**Authors:** Qi-He Tang, Wan-Li Li, Jie-Ping Wang, Xi-Jie Li, Dan Li, Zhe Cao, Qi Huang, Jia-Li Li, Jun Zhang, Zheng-Wei Wang, Jun Guo, Ji-Lian Li

**Affiliations:** ^1^ Key Laboratory for Insect-Pollinator Biology, Ministry of Agriculture and Rural Affairs, Institute of Apicultural Research, Chinese Academy of Agricultural Sciences, Beijing, China; ^2^ Faculty of Life Science and Technology, Kunming University of Science and Technology, Kunming, China; ^3^ ChongQing Academy of Animal Sciences, Chongqing, China; ^4^ CAS Key Laboratory of Tropical Forest Ecology, Xishuangbanna Tropical Botanical Garden, Chinese Academy of Sciences, Jinghong, China

**Keywords:** bumblebees, glyphosate (GLY), spinetoram, sublethal effects, gut microbes, physiological biomarkers

## Abstract

The sublethal effects of pesticide poisoning will have significant negative impacts on the foraging and learning of bees and bumblebees, so it has received widespread attention. However, little is known about the physiological effects of sublethal spinetoram and glyphosate exposure on bumblebees. We continuously exposed *Bombus terrestris* to sublethal (2.5 mg/L) spinetoram or glyphosate under controlled conditions for 10 days. The superoxide dismutase, glutathione-S-transferase, carboxylesterase, prophenoloxidase, α-amylase and protease activities, and changes in gut microbes were measured to understand the effects of sublethal pesticide exposure on the physiology and gut microbes of bumblebees. Sublethal pesticide exposure to significantly increased superoxide dismutase activity and significantly decreased gut α-amylase activity in bumblebees but had no significant effect on glutathione-S-transferase, carboxylesterase or gut protease activities. In addition, glyphosate increased the activity of prophenoloxidase. Interestingly, we observed that neither of the two pesticides had a significant effect on dominant gut bacteria, but glyphosate significantly altered the structure of the dominant gut fungal community, and reduced the relative abundance of *Zygosaccharomyces* associated with fat accumulation. These results suggest that sublethal spinetoram and glyphosate do not significantly affect the detoxification system of bumblebees, but may affect bumblebee health by inhibiting energy acquisition. Our results provide information on the sublethal effects of exposure to low concentrations of glyphosate and spinetoram on bumblebees in terms of physiology and gut microbes.

## 1 Introduction

Bumblebees are important pollinators of crops and wild plants. Their pollination of European crops is valued at more than 22 billion euros each year and is vital to food security (Nieto et al., 2014; Tasman et al., 2020). In addition, because bumblebees are better at pollinating crops that do not produce nectar than honeybees, they are often used commercially in Asia for pollination of greenhouse crops such as tomatoes (Kokuvo et al., 2008; Takeuchi et al., 2018). However, the populations of many bumblebee species are declining dramatically (Tsvetkov et al., 2021). These declines are thought to be caused by a combination of factors, such as habitat loss, pathogen infestation, invasive species, and climate change, with pesticide use being considered a serious contributing factor to wild bee declines (Baron et al., 2017; Soroye et al., 2020).

Pesticides have the disadvantages of toxicity, non-selective action and bioaccumulation potential (Gierer et al., 2019, Murawska et al., 2021). Therefore, they can have negative effects on non-target organisms such as social bees (Di Noi et al., 2021). After pesticides are used in agriculture, they remain in the nectar and pollen of plants and may be collected by bees and brought back to the hive. Recently, there have been many studies reporting the presence of pesticides in hives (Jabot et al., 2015; Wen et al., 2021; Demares et al., 2022; Perez-Cobo et al., 2022; Xiao et al., 2022). However, pesticide exposure levels in the hives are usually below the levels that directly cause bee mortality. For example, Farooqi et al. (2017) tested honey samples from Pakistan for pesticide residues from 2013 to 2014 and found that the highest residue concentration of deltamethrin was .023 mg/kg, while the 48 h LC50 for deltamethrin was 60.8 mg/L (Dai et al., 2010). Therefore, the bees will be continuously exposed to the sublethal pesticides, and exposure to sublethal pesticides can have a range of negative effects on foraging, learning and endocrine system functioning (Gill and Raine, 2014; Christen et al., 2018; Smith et al., 2020).

Spinetoram is a semisynthetic insecticide obtained by synthetic modification of spinosyn biological insecticides (Betz and Andrew, 2020). It acts on synaptic transmission (Piovesan et al., 2020), and studies have shown that exposure to high levels of spinetoram threatens the survival of bumblebees (Besard et al., 2011). Glyphosate is an organophosphorus herbicide (Green, 2016; Weidenmuller et al., 2022). Since the target enzyme of glyphosate, 5-enolpyruvoylshikimate 3-phosphate synthase (EPSPS, which is an essential enzyme for plants, fungi and some bacteria), is not found in insects (Schonbrunn et al., 2001; Weidenmuller et al., 2022). Glyphosate was thought to be harmless to insects such as bumblebees and honeybees for many years (Thompson et al., 2014; Odemer et al., 2020). However, recent studies have found that honeybee exposure to glyphosate has non-target sublethal effects on reproductive capacity, immunity, and cognitive ability in honeybees (Chmiel et al., 2020; Odemer et al., 2020). Moreover, Weidenmuller et al. (2022) found that consistent glyphosate exposure impairs the thermoregulatory capacity of bumblebees. Worryingly, 4,000 kg of honey harvested in the German state of Brandenburg in 2020 was found to be heavily polluted with glyphosate at levels of up to 76 mg/kg (Odemer et al., 2020). This suggests that bees may be continuously exposed to glyphosate in the hive environment. However, there are few other reports of sublethal consistent exposure to spinetoram or glyphosate damaging the health of bumblebees.

The antioxidant and detoxification mechanisms of honeybees are triggered when the bees are exposed to sublethal concentrations of pesticides (Li et al., 2017); superoxide dismutase (SOD) is an important antioxidant protein in insect antioxidant mechanisms (Wang W. L. et al., 2019). Glutathione-S-transferase (GST) and carboxylesterase (CarE) are the main enzymes involved in the detoxification process in insects (Berenbaum and Johnson, 2015; Li et al., 2017). In addition, a number of other enzymes are important in the physiological processes of insects. For example, innate immune proteins engaged in cellular and humoral defenses, such as prophenoloxidase (PPO) (Wang Y. F. et al., 2019), and α-amylase and protease, are involved in gut digestive processes (Kilani-Morakchi et al., 2017; Trestrail et al., 2021), but the toxic effects of continuous sublethal spinetoram and glyphosate exposure on organisms are unclear. Using enzyme activity as an index for toxicological studies can improve our knowledge of the toxic effects of sublethal spinetoram and glyphosate exposure. These enzyme activities in honeybees after exposure to pesticides have been widely studied (Murawska et al., 2021), but to our knowledge there have been no studies on the effects of exposure to spinetoram and glyphosate on these enzymes in bumblebees.

Gut microbes are critical to host health (Zhu L. Z. et al., 2020), and perturbation of the gut microbial community can induce host phenotypic changes related to metabolism, development and immunity and even affect the memory behavior of bumblebees (Liu et al., 2020; Li et al., 2021). Thus, disruption of gut microbial homeostasis can indirectly affect the health of the bees. Exposure to pesticides constitutes a significant threat to the structure and composition of the gut microbial community in bees (Yang et al., 2019). However, the effects of spinetoram and glyphosate on bumblebee gut microbes are not clear.

To fill these knowledge gaps, in this study, we exposed *Bombus terrestris* workers to low concentrations of spinetoram and glyphosate under laboratory conditions for 10 days, respectively. Multiple indicators were measured for the workers to evaluate the effects of spinetoram and glyphosate on (1) survival; (2) SOD, GST, CarE and PPO activity in the body; (3) gut α-amylase and protease activity; (4) gut bacterial homeostasis; and (5) gut fungal homeostasis. These results provide insight on the effects of sublethal spinetoram and glyphosate exposure on *B. Terrestris* from physiological and gut microbial perspectives, as well as provide a basis for studying the effects of pesticides on the physiological indicators and gut microbes of bumblebees.

## 2 Methods and materials

### 2.1 Acquisition of worker bees

To obtain *Bombus terrestris* workers of similar size and age, *B. Terrestris* queens were reared in small plastic cages in the dark at a temperature of 28°C and relative humidity of 50%–60% at the Institute of Apicultural Research, Chinese Academy of Agricultural Sciences, Beijing, China. Sugar water (50%, wt/vol) and pollen were provided *ad libitum* to the 20 colonies that were subsequently produced until males and gynes emerged. Then, healthy adult *B. Terrestris* workers of a similar size and age were selected for subsequent experiments.

### 2.2 Toxicity bioassay

This experiment was conducted from late July to early August 2021. Glyphosate ammonium salt granules (equivalent to 30% of the acid glyphosate) were purchased from Zhejiang New Anhua Group Co., Ltd., Hangzhou, China. The Dow AgroSciences formulation of spinetoram suspension concentrate (effective content of 60 g/L) was purchased from a vendor. Both pesticides were diluted with sucrose syrup (50%, wt/vol) to 20 mg/L, 10 mg/L, 5 mg/L, and 2.5 mg/L (active ingredient content). Then, the prepared pesticide syrups were sterilized using filtration.

A total of 90 adult worker bees were selected and divided into 9 groups, and each group was randomly assigned 10 worker bees to the same plastic cup. The workers were acclimatized for 1 h in a dark environment at 28°C–30°C and 50%–55% relative humidity (Besard et al., 2011; Farder-Gomes et al., 2021). Then, four groups of workers were fed different concentrations (20 mg/L, 10 mg/L, 5 mg/L, and 2.5 mg/L) of spinetoram syrup, four groups were fed different concentrations (20 mg/L, 10 mg/L, 5 mg/L, and 2.5 mg/L) of glyphosate syrup, and one group was fed filter-sterilized normal syrup (50%, wt/vol). The number of worker deaths was observed and recorded each day until the end of the experiment on day 10. All bees were fed *ad libitum* during the experiment. This experiment was repeated three times independently.

### 2.3 Sample treatment and collection

**FIGURE 1:**
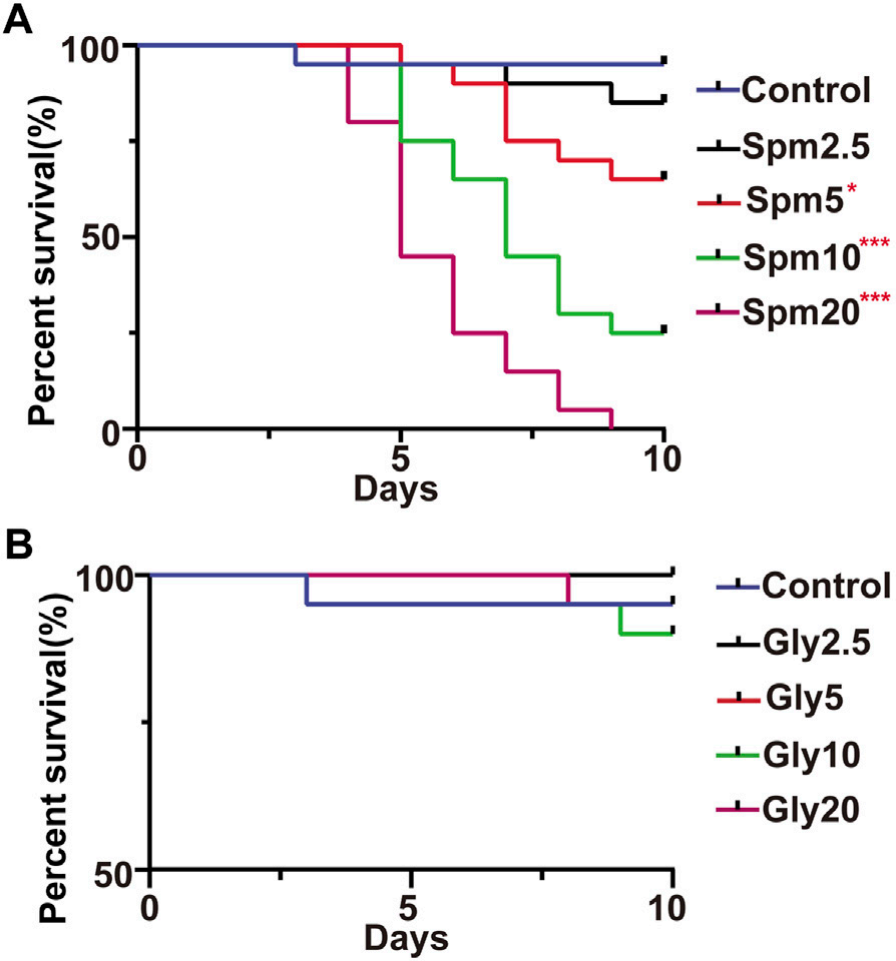
Effects of spinetoram **(A)** and glyphosate **(B)** on the survival of bumblebees. Spm indicates the spinetoram-exposed group, Gly indicates the glyphosate-exposed group, and the numbers indicate the concentration (mg/L). Statistical analysis of the treatment and control groups was performed using the log-rank test (*indicates .05 > *p* > .01; ** indicates .001 < *p* < .01; *** indicates *p* < .001, *n* = 10).

As shown in Figure 1, we selected 2.5 mg/L glyphosate and 2.5 mg/L spinetoram as the subsequent experimental groups. Ten days after the oral toxicity experiment, five worker bees were randomly selected in each parallel experiment from the normal syrup group (Control), the low-concentration (2.5 mg/L) spinetoram group (Spm2.5), and the low-concentration (2.5 mg/L) glyphosate group (Gly2.5). These worker bees were made unconscious by placing them in a freezer at −20°C for 20 min. The brain and thorax tissues of the workers were dissected according to the method reported by Li et al. (2017).Chitin from the brain and thorax of the worker bees was removed and mixed together for each group of five workers. Then, 1.0 mL of phosphate-buffered saline (PBS) was added to the sample for each group and well homogenized. The homogenate was centrifuged at 4°C and 14,000 × g for 10 min, and the supernatant was collected. The mixed body homogenate of worker bees was used for subsequent analysis or stored at −80°C. In addition, the intestines extracted from each of the five groups were mixed together (Zhang et al., 2019), homogenized with 500 μL of PBS, divided into two parts (100 μL for digestive enzyme activity determination and the remaining for microbial diversity analysis), and stored immediately at −80°C for subsequent analysis.

### 2.4 Measurement of physiological biomarkers

The total protein concentrations in the mixed body homogenate and the gut homogenate were determined using the Pierce@ BCA Protein Assay Kit (Thermo Scientific, Waltham, MA, United States) according to the manufacturer’s instructions.

#### 2.4.1 Antioxidant, detoxification and immune enzymes

The ELISA technique was used to quantify SOD, GST, CarE and PPO in the body homogenate. SOD, GST, CarE and PPO concentrations in the mixed body tissues of workers were measured using ELISA kits (Shanghai Enzyme-linked Biotechnology Co., Ltd., Shanghai, China) according to the manufacturer’s instructions.

#### 2.4.2 Digestive enzymes

α-Amylase activity was assayed by the dinitrosalicylic acid (DNS) procedure, using 1% soluble starch as substrate (Kilani-Morakchi et al., 2017). Five microliters of the gut homogenate was incubated for 60 min at 35°C with 100 μL of PBS (pH 7.2) and 95 μL of substrate. Then, 100 µL of DNS was added, and the sample was heated in boiling water for 10 min to stop the reaction. After cooling at room temperature, 100 μL of distilled water was added to the reaction solution and shaken well, and the absorbance at 540 nm was measured immediately. The blank solution was prepared by the same method, but the gut homogenate was heated at 90°C for 10 min to inactivate the enzyme. One milligram/mL maltose was used as a standard solution. A standard curve (y = 1.6337x+.0058) was established using different concentrations of maltose (0–1 mg/mL). One unit (U) of α-amylase activity was defined as the amount of enzyme required to produce 1 mg of maltose in 60 min at 35°C.

Protease activity was determined according to the method reported by Trestrail et al. (2021). First, 10 µL of gut homogenate was mixed with 40 µL of casein containing 1% (w/v). After 60 min of reaction at 37°C, 50 µL of .4 M trichloroacetic acid solution was added to terminate the reaction. The reaction was then centrifuged at ×10,000 g for 10 min at 4°C, and the supernatant was collected. Fifty microliters of the supernatant was mixed with 250 μL of .4 M sodium bicarbonate solution and 50 μL of forintanol reagent, and the absorbance at 660 nm was measured after color development at 37°C for 20 min. The blank solution was prepared by the same method, but the gut homogenate was treated with 90°C for 10 min to inactivate the enzyme. L-tyrosine (100 μg/mL) was used as a standard solution. A standard curve (y = 3.0375x–.0053) was established using different concentrations of L-tyrosine (0–100 μg/mL). One unit (U) of protease activity was defined as the amount of enzyme required to produce 1 mg of L-tyrosine in 20 min at 37°C.

### 2.5 Gut microbial analysis

Total gut microbial genomic DNA was extracted using an E.Z.N.A. Soil DNA Kit (Omega Bio-Tek, United States). The quantity and quality of the DNA were measured using a NanoDrop 2000 UV–vis spectrophotometer (Thermo Scientific, Wilmington, United States) and 1.0% agarose gel electrophoresis. The variable V3 and V4 regions of the bacterial 16S rRNA gene were amplified with the primers 338F (5’-ACT​CCT​ACG​GGA​GGC​AGC​AG-3’) and 806R (5’-GGACTACHVGGGTWTCTAAT-3’) (Tang et al., 2021). The ITS1-ITS2 region of fungal ITS genes was amplified with the primers ITS1 (5’-CTT​GGT​CAT​TTA​GAG​GAA​GTA​A-3’) and ITS2 (5’-GCT​GCG​TTC​TTC​ATC​GAT​GC-3’) (Disayathanoowat et al., 2020). Quality checking of PCR products was performed by 2% agarose gel electrophoresis. Sequencing libraries were created with the instructions provided with the TruSeqTM DNA Sample Prep Kit (Illumina, SD, United States). The libraries were mixed and paired-end sequenced on an Illumina MiSeq platform (Illumina, United States) according to the standard operating instructions. The raw data for the 16 S rRNA and ITS genes were filtered by QIIME 1.7 and merged using FLASH 1.2 operational taxonomic unit (OTU) clustering with 97% similarity using UPARSE 7.0. The taxonomy of the OTUs was analyzed by the RDP classifier algorithm, and a confidence threshold of 70% was used with the SILVA database for 16S rRNA and the fungal ITS database for comparison (Dong et al., 2021; Song et al., 2021). The complete data were deposited in the NCBI Sequence Read Archive (SRA) database (accession numbers: SRR 18693273-18693281 and SRR 18693405-18693410).

### 2.6 Statistical analysis

The differences between the survival curves of each treatment group and the control group were compared using the log-rank test, which is a built-in method in GraphPad Prism 8.3. The activity of each enzyme in specific tissues of workers is expressed as specific activity, where specific activity = enzyme activity/total protein content. The Dunnett test (SPSS 19.0 for Windows) was used to compare the significant differences between the control group and the other groups. For gut fungal analysis, the independent samples t-test (SPSS 19.0 for Windows) was used to calculate the significant differences between the two groups. The group comparison for beta diversity was performed using permutational multivariate analysis of variance (PERMANOVA) and analysis of similarities (ANOSIM) in the vegan package of R 3.3 software.

## 3 Results

### 3.1 Oral toxicity test

There was no significant difference in survival between workers in the control group and those that ingested 2.5 mg/L spinetoram during the 10 days of the experiment (*p* > .05, Figure 1A). However, the survival of worker bees in the control group was significantly higher than that of workers in the groups that ingested 5–20 mg/L spinetoram syrup (*p* < .001, Figure 1A). The results showed that higher concentrations of spinetoram syrup were toxic to adult workers. Interestingly, there was no survival effect of any glyphosate concentration tested (*p* > .05, Figure 1B). Therefore, 2.5 mg/L glyphosate or spinetoram was within the range of sublethal concentrations for *B. terrestris* workers.

### 3.2 Antioxidant, detoxification and immune enzyme activity changes

**FIGURE 2:**
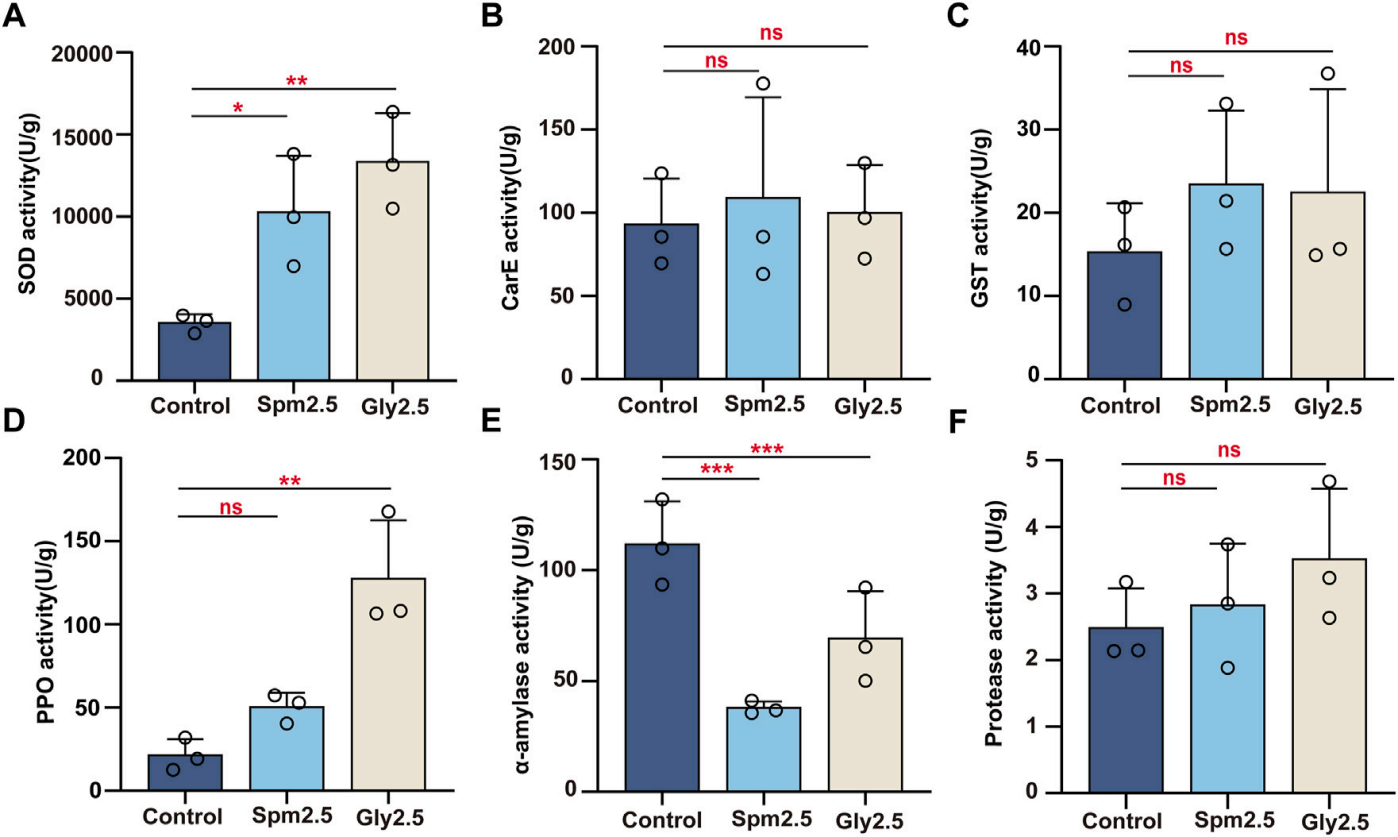
Effects of chronic sublethal spinetoram (Spm2.5) and glyphosate (Gly2.5) exposure on superoxide dismutase **(A)**, carboxylesterase **(B)**, glutathione-S-transferase **(C)**, prophenoloxidase **(D)**, α-amylase **(E)** and protease **(F)** activities in bumblebees. Statistical analyses were performed using the Dunnett test (* indicates .05 > *p* > .01; ** indicates .001 < *p* < .01; *** indicates *p* < .001, *n* = 3).

We examined the changes in SOD, CarE, GST and PPO activities in the mixed body tissues of workers after 10 days of exposure to sublethal concentrations (2.5 mg/L) of spinetoram and glyphosate (Figures 2A–D). Exposure to spinetoram significantly increased SOD activity (*p* = .035) in the mixed body tissues of workers 1.9-fold, but the differences in CarE (*p* = .507), GST (*p* = .860) and PPO (*p* = .249) activities were non-significant. Similarly, exposure to glyphosate significantly increased SOD (*p* = .007) and PPO (*p* = .004) activities in the mixed body tissues of workers 2.8-fold and 5.0-fold, respectively, compared to those in control workers, but the differences in CarE (*p* = .972) and GST (*p* = .579) activities were non-significant.

### 3.3 Gut digestive enzyme changes

For gut α-amylase activity, both exposure to sublethal spinetoram and glyphosate significantly decreased their activities by 66.11% (*p* = .003) and 38.03% (*p* = .036), respectively, compared to the control group (Figure 2E). For gut protease activity, no significant difference was found between the control and spinetoram-treated groups (*p* = .854) or between the control and glyphosate-treated groups (*p* = .321) by Dunnett’s test (Figure 2F).

### 3.4 Response of bumblebee gut bacteria to sublethal exposure to pesticides

High-throughput sequencing of the gut bacterial community of bumblebees was performed. After initial quality control, a total of 649,409 high-quality sequences with an average read length of 427 bp were obtained from 9 gut samples. In all samples, the sampling depth was normalized based on the lowest number of read sequences (value = 35,316), and 32 OTUs were obtained. The OTU% of the samples was estimated using Good’s coverage index, and the bacterial coverage exceeded 99.98% in all samples (Supplementary Table S1), indicating that the natural bacterial diversity was well covered and that the database was sufficient to analyze the dominant bacterial community information.

**FIGURE 3:**
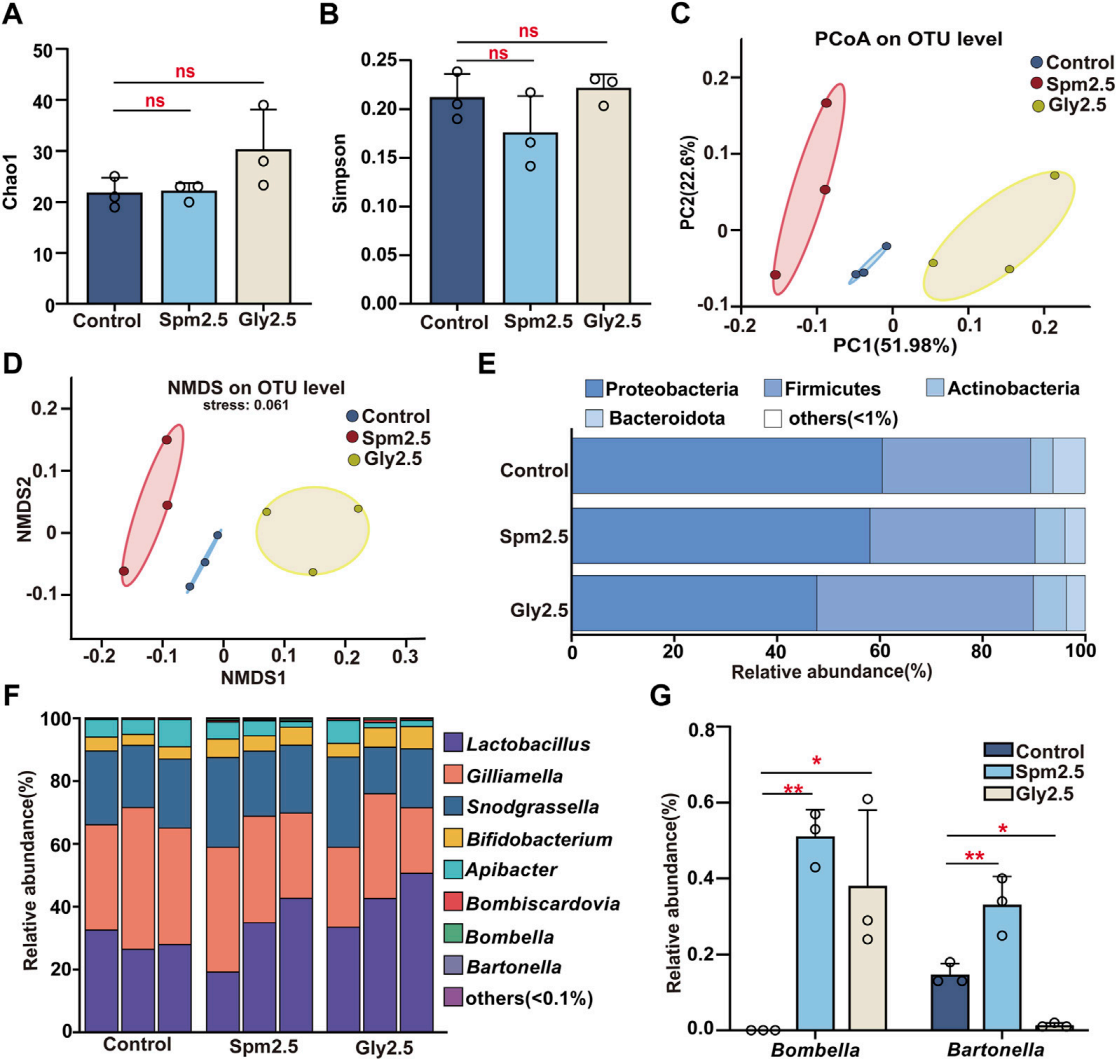
Effects of chronic sublethal spinetoram (Spm2.5) and glyphosate (Gly2.5) exposure on gut bacteria in bumblebees. **(A)** Sobs index. **(B)** Shannon index. **(C)** Principal coordinates analysis (PCoA, based on Bray-Curtis distance). **(D)** Non-metric multidimensional scaling (NMDS, based on Bray-Curtis distance). **(E)** Relative abundances of dominant bacterial phyla. **(F)** Bacterial genera with relative abundances greater than .1%. **(G)** Genera with significant differences in relative abundance. Statistical analyses were performed using the Dunnett test (* indicates .05 > *p* > .01; ** indicates .001 < *p* < .01, *n* = 3).

The results of gut alpha diversity bacterial analysis showed (Figures 3A, B; Supplementary Table S1) that neither spinetoram exposure nor glyphosate exposure caused significant changes in the community richness and bacterial community evenness of worker gut bacteria compared to those of the control group(*p* > .05). Based on Bray-Curtis distances, both principal coordinate analysis (PCoA) and non-metric multidimensional scaling (NMDS) showed (Figures 3C, D) that the three groups of samples were clustered into three taxa, which could be clearly distinguished among the different treatment groups. Significant differences in the gut bacterial communities of these three groups were found by analysis of similarities (*p* = .001, 999 permutations in each test).

Phyla with relative abundances greater than 1% in the three groups of samples were described (Figure 3E), and these included Proteobacteria, Firmicutes, Actinobacteria and Bacteroidota. Compared to the control group, neither spinetoram exposure nor glyphosate exposure caused significant changes in the relative abundances of these dominant bacterial phyla in bumblebees (*p* > .05, Supplementary Figure S1A). We similarly mapped the major bacterial genera with relative abundances greater than .1%, which were *Lactobacillus*, *Gilliamella*, *Snodgrassella*, *Bifidobacterium*, *Apibacter*, *Bombiscardovia*, *Bombella* and *Bartonella* (Figure 3F). Among them, the relative abundances of *Lactobacillus*, *Gilliamella*, *Snodgrassella*, *Bifidobacterium*, and *Apibacter* all exceeded 1%, and the relative abundances of these five bacterial genera exceeded 98.5% in each sample, so they were considered the core bacterial genera of the three groups. Notably, there was no significant difference in the relative abundances of these core bacterial genera in either the spinetoram exposure group or the glyphosate exposure group compared to the control group (*p* > .05, Supplementary Figure S1B). However, the relative abundances of some non-core bacterial genera (such as *Bombella* and *Bombella*) were found to be significantly altered in the spinetoram- or glyphosate-exposed groups compared to the control group (*p* < .05, Figure 3G).

### 3.5 Response of bumblebee gut fungi to sublethal glyphosate exposure

A quality check of the fungal PCR products was performed by 2% agarose gel electrophoresis, in which the results of the quality check of the spinetoram group were unacceptable. Therefore, only the ITS1-ITS2 region of the ITS gene of the gut fungi in the control and glyphosate exposure groups was subjected to high-throughput sequencing. After quality control and removal of unclassified fungi, 49,968 high-quality sequences per sample with an average length of 277 bp were acquired from six samples. These sequences were grouped into 179 OTUs based on a sequence similarity higher than 97%. In addition, the OTU% of the samples was estimated using Good’s coverage index, and the bacterial coverage exceeded 99.98% in all samples (Supplementary Table S2), indicating that the natural fungal diversity was well covered and that the database was sufficient to analyze the dominant fungal community information.

**FIGURE 4:**
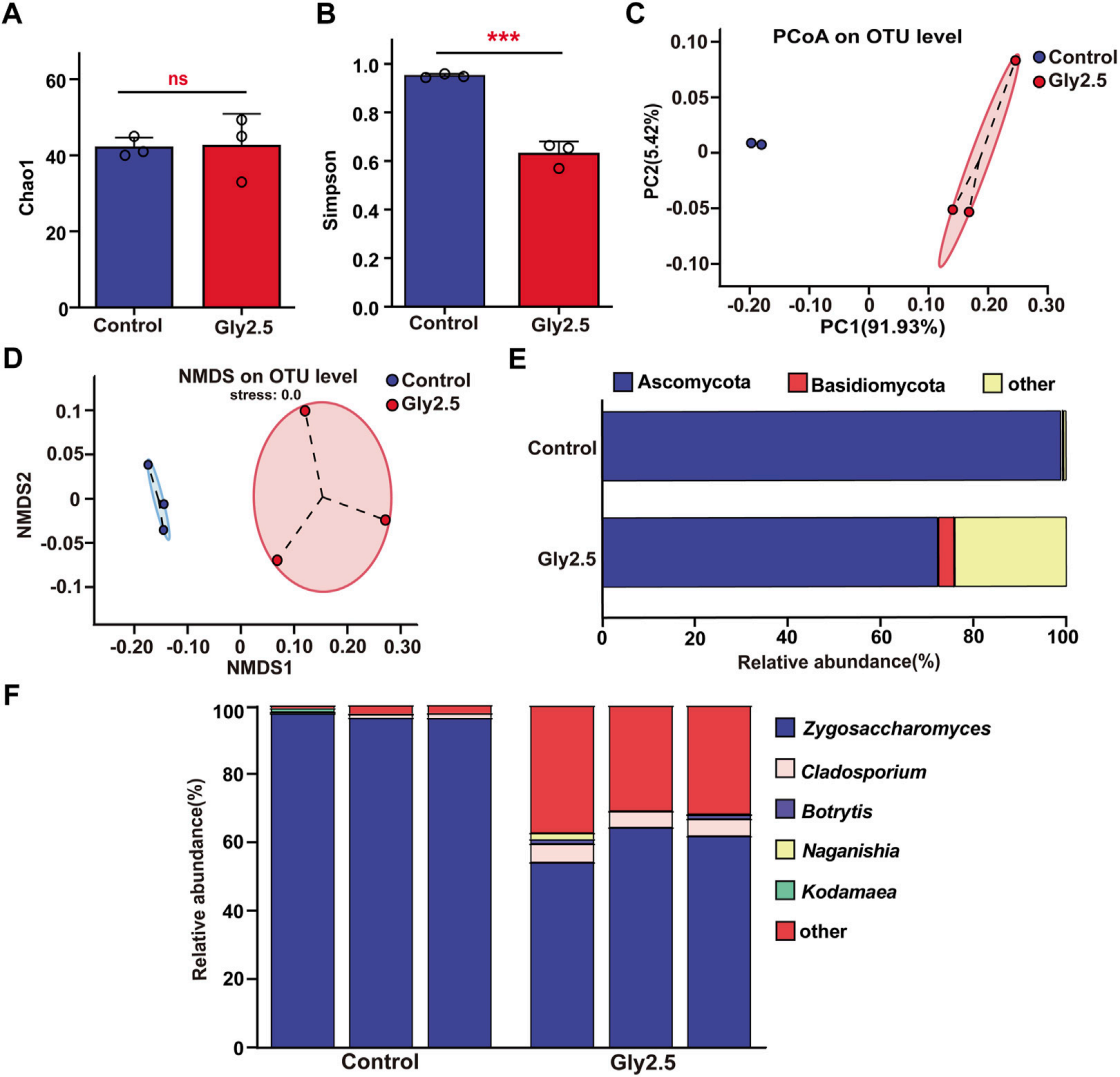
Effects of chronic sublethal glyphosate (Gly2.5) exposure on gut fungi in bumblebees. **(A)** Sobs index. **(B)** Shannon index. **(C)** PCoA based on Bray-Curtis distance. **(D)** NMDS analysis based on Bray-Curtis distance. **(E)** Relative abundances of dominant fungal phyla. **(F)** Relative abundances of dominant fungal genera. Statistical analyses were performed using Student’s t-test (*** indicates *p* < .001, *n* = 3).

The results of gut alpha diversity fungi indicated (Figures 4A, B; Supplementary Table S2) that glyphosate exposure significantly increased fungal community evenness compared to that of the control (*p* < .001) but had no significant effect on fungal community richness (*p* = .449). PCoA and NMDS showed (Figures 4C, D) that the two groups of samples clustered into two taxa, which could be clearly distinguished between the control and glyphosate-treated groups. This result suggests that glyphosate significantly altered the structure of the gut fungal community.

At the phylum level, the relative abundances of Ascomycota and Basidiomycota in the two groups of samples were greater than 1% (Figure 4E). After glyphosate exposure, the relative abundance of Ascomycota (*p* < .001) decreased significantly from 98.84% to 72.34% and that of Basidiomycota (*p* = .039) increased significantly from .45% to 3.56% (Supplementary Table S3). At the genus level, we mapped the dominant fungal genera with relative abundances higher than 1% in one of the samples, which were *Zygosaccharomyces*, *Cladosporium*, *Botrytis*, *Naganishia* and *Kodamaea* (Figure 4F). Notably, the composition of dominant gut fungi in the control group was highly conserved, with *Zygosaccharomyces*, whose relative abundance reached 96.83%, as the only genus with a relative abundance greater than 1%, while the relative abundance of *Cladosporium*, the second most abundant genus, was only .95%. Glyphosate exposure increased the diversity of dominant fungi in the gut, with the relative abundance of both *Zygosaccharomyces* (60.14%) and *Cladosporium* (5.05%) being greater than 1%, and the relative abundance of the two fungal genera was significantly different compared to the control group from that of the control (*p* < .001, Supplementary Table S3). These results suggest that glyphosate significantly altered the composition of the gut fungal community.

## 4 Discussion

In this study, we explored the sublethal effects of separate exposure to spinetoram and glyphosate on the physiological biomarker enzymes of bumblebees (*Bombus terrestris*), confirming that sublethal (2.5 mg/L) exposure to spinetoram and glyphosate impairs the health status of bumblebees by causing oxidative stress and inhibiting gut α-amylase activity. Moreover, through high-throughput sequencing techniques, we found that although spinetoram and glyphosate had no significant effects on the abundance of core gut bacteria, glyphosate significantly altered the abundance of core gut fungi.

Zhu Y. C. et al. (2020) showed that the soluble protein content of honeybees gradually decreases with the increase of honeybee age, and the sensitivity to pesticides gradually increases. Therefore, in this study we conducted oral toxicity experiments using worker bees of a similar age. Our results showed that spinetoram exposure significantly reduced the survival of *B. terrestris* in a dose-dependent manner under controlled conditions, and this result was supported by Besard et al. (2011). Notably, in this study, consistent exposure (10 days) to 2.5 mg/L spinetoram did not cause mortality. Therefore, we believe that 2.5 mg/L spinetoram was a sublethal concentration for *B. terrestris* workers. Similar to our results, Herbert et al. (2014) found that exposure (14 days) of honeybees to 2.5 mg/L and 5 mg/L glyphosate did not cause significant worker mortality. However, some studies have pointed out that exposing (24 h) honeybees and bumblebees to commercial products containing glyphosate preparations, such as Roundup®, can cause a significant increase in their mortality (Motta et al., 2020; Straw et al., 2021). By exposing *B. terrestris* to Roundup® containing 7.2 g/L glyphosate, non-glyphosate Roundup® and Weedol® containing 7.2 g/L glyphosate (.02 g/L pyraflufen-ethyl), Straw et al. (2021) found no significant increase in mortality of bumblebees exposed (24 h) to Weedol®, while mortality increased by 96% under non-glyphosate Roundup® (24 h). This confirms that bee mortality caused by Roundup® is due to the toxic active agent it contains and not glyphosate, which provides support for our results that consistent exposure (10 days) to 2.5–20 mg/L glyphosate didn't cause significant mortality in *B. terrestris* workers.

After exposure to pesticides, insects usually experience oxidative stress in their bodies, generating free radicals (Abdelsalam et al., 2020) and causing oxidative stress damage, while SOD has the ability to play a role in eliminating free radicals (Zhang et al., 2020). In the present study, exposure to glyphosate or spinetoram significantly increased SOD activity in tissues compared to the control group. This suggests that consistent sublethal exposure to spinetoram or glyphosate induces an oxidative stress response in the bumblebee and requires activation of SOD enzymes to mitigate oxidative stress damage. Detoxification enzymes are part of the physiological defense of insects, which metabolize many exogenous toxins (AlJabr et al., 2017; Jiang et al., 2021). The CarE enzyme is mainly responsible for inactivating toxins by changing their structure and rendering them inactive, while GST is mainly involved in the coupling of detoxification products for solubilization and transport (Berenbaum and Johnson, 2015; Li et al., 2017). However, neither CarE nor GST activity was altered in the bumblebee after exposure to spinetoram or glyphosate. This suggests that bumblebees cannot detoxify these two pesticides on their own or that other physiological detoxification mechanisms, such as P450 (Daborn et al., 2002), exist in bumblebees. Interestingly, exposure to glyphosate seems to induce an innate immune response in bumblebees. This is because consistent sublethal exposure to glyphosate enhances the activity of PPO, an enzyme that is an essential natural immune protein for insect cellular and humoral defense (Wang Y. F. et al., 2019). The relationships between antioxidant, detoxification and immune enzymes and pesticide toxicity in bumblebees are quite complex, and further studies are needed to fully understand them.

Amylase is an important enzyme for energy synthesis and digestion in insects and directly impacts the uptake and utilization of nutrients (Cai et al., 2021). Our results indicate that consistent sublethal exposure to spinetoram or glyphosate significantly reduced gut amylase activity. This effect may impair bumblebee health by reducing bumblebee digestion and body weight. Although specific data on the effects of pesticides on bumblebee body weight are lacking, Liu et al. (2020) used the pesticide thiacloprid in honeybees and found it to cause a decrease in bee body weight. Worryingly, the reduction in insect gut amylase activity by pesticide exposure seems to be universal. For example, Abo El-Ghar et al., 1995 showed that sublethal concentrations of a pesticide (abamectin) reduced amylase activity in *Spodoptera littoralis* larvae. Vyjayanthi and Subramanyam (2002) also found that pesticide (fenvalerate) exposure reduced amylase activity in the midgut of *Bombyx mori* larvae. Therefore, we need to pay more attention to the negative effects of these changes in gut digestive enzymes on bumblebees in future studies.

Similar to previous findings, we found that Proteobacteria, Firmicutes, Actinobacteria and Bacteroidetes were the main phyla of bumblebee gut bacteria (Koch and Schmid-Hempel, 2011); *Lactobacillus*, *Gilliamella*, *Snodgrassella*, *Bifidobacterium* and *Apibacter* were the main genera of bumblebee gut bacteria (Hammer et al., 2021). Interestingly, we found that the relative abundance of core gut bacteria in bumblebees did not change significantly after prolonged exposure to glyphosate or spinetoram at either the phylum level or the genus level. Recently, Almasri et al. (2022) also observed this phenomenon. They used low concentrations of three pesticides, namely, imidacloprid, glyphosate and difenoconazole, and a ternary mixture for exposure (9 days) of honeybees and found that exposure to these pesticides did not affect the core bacteria of the honeybee gut. However, Motta et al. (2018) found that short-term glyphosate exposure altered the composition of gut bacteria in bees. Notably, Liu et al. (2020) found that while bees exposed to thiacloprid for a short period had a disturbed gut bacterial community, consistent (13 days) exposure had a restorative effect on the bacterial community. Therefore, alterations in the bacterial community were not found in our results, which may also have been caused by the limitations of sequencing sampling time points.

In addition to containing a large number of gut bacteria, the bee gut is rich in fungi, which have antibacterial and antioxidant activities, and probiotic potential (Hsu et al., 2021; Cui et al., 2022; Rutkowski et al., 2022). However, the effects of pesticide exposure on the gut fungi of bees are often overlooked. In this study, the effects of exposure to low concentrations of glyphosate on bumblebee gut fungal populations were investigated using ITS amplicon sequencing. We found that the gut fungal community of all the bumblebees was dominated by Ascomycota at the phylum level and by *Zygosaccharomyces* at the genus level, which is consistent with previous findings on gut fungi in *A. mellifera* queens (Yun et al., 2018). Sublethal glyphosate exposure significantly altered the relative abundances of a few key core fungal genera in the bumblebee gut. For example, the *Zygosaccharomyces* abundance in the gut was reduced by the presence of glyphosate compared to that in the control bumblebees. According to a recent study (Canche-Colli et al., 2021), *Zygosaccharomyces* is associated with the accumulation of fat bodies in bees. The researchers supplemented food with *Zygosaccharomyces mellis*, allowing bees to consume more food and metabolize excess nutrients, thereby increasing the accumulation of fat bodies in *A. mellifera*. Therefore, the decrease in the abundance of *Zygosaccharomyces* caused by glyphosate exposure may affect the accumulation of fat bodies in bumblebees, thus affecting their weight gain. The effect of glyphosate on weight loss in bees has been studied in *A. mellifera* larvae (Vazquez et al., 2018). Therefore, we believe that sublethal glyphosate exposure alters the structure and composition of the bumblebee gut fungal community and thus may negatively affect bumblebee growth and development. However, as mentioned earlier, the duration of pesticide exposure (sampling time) varies and affects the composition of the gut microbes. Therefore, more studies are needed to confirm this conclusion.

Previous studies have shown that while sublethal pesticide exposure does not directly kill honeybees, it can cause disorientation and memory loss in bees, and reduce the efficiency of bumblebee pollen collection and other foraging properties (Gill et al., 2012; Gill and Raine, 2014; Chmiel et al., 2020), thus having significant impacts on bees and bumblebees. Our study showed that under controlled conditions, 10-day exposure to high concentrations (5–25 mg/L) of spinetoram significantly reduced the survival of *B. terrestris*, whereas exposure to 2.5–20 mg/L glyphosate or 2.5 mg/L spinetoram didn't have such an effect. However, exposure to sublethal spinetoram and glyphosate caused activation of SOD activity by oxidative stress and significantly reduced α-amylase activity in the gut, respectively. Furthermore, glyphosate significantly and selectively reduced the relative abundance of core fungi (*Zygosaccharomyces*) associated with fat accumulation. Therefore, we conclude that exposure to sublethal spinetoram and glyphosate, although unlikely to kill bumblebees, could change physiological processes and gut microbes related to oxidative stress, food digestion, and energy accumulation, thereby affecting bumblebee health. Since bees often bring nectar and pollen containing pesticides back to the nest, every bee in the nest is exposed, even the larvae. In addition, gut microbes can also affect the memory of bumblebees (Li et al., 2021). Therefore, subsequent studies should consider the actual effects of these two pesticides on different individual bumblebees in the hive environment, and whether the pesticides can alter bumblebee memory, foraging, and other behaviors by affecting bumblebee gut microbes.

## Data Availability

The datasets presented in this study can be found in online repositories. The names of the repository/repositories and accession number(s) can be found below: https://www.ncbi.nlm.nih.gov/, SRR 18693273-18693281 and SRR 18693405-18693410.

## Supplementary material

SUPPLEMENTARY FIGURE S1 Effects of chronic 2.5 mg/L glyphosate (Gly) and 2.5 mg/L spinetoram (Spm) on the dominant bacterial phylum **(A)** and bacterial genus **(B)** in the gut of the bumblebee. Statistical analyses were performed using the Dunnett test (ns indicates *p* > .05).

## References

[B1] Abdelsalam S. Alzahrani A. M. Elmenshawy O. M. Abdel-Moneim A. M. (2020). Antioxidant status and ultrastructural defects in the ovaries of red palm weevils (*Rhynchophorus ferrugineus*) intoxicated with spinosad. Entomol. Res. 50, 309–316. 10.1111/1748-5967.12442

[B2] Abo El-Ghar G. E. S. Radwan H. S. A. El-Bermawy Z. A. Zidal T. M. (1995). Inhibitory effect of thuringiensin and abamectin on digestive enzymes and non-specific esterase of *Spodoptera littoralis* (Boisd) (Lep., Noctuidae) larvae. J. Appl. Entomol. 119, 355–359.

[B3] AlJabr A. M. Hussain A. Rizwan-ul-Haq M. Al-Ayedh H. (2017). Toxicity of plant secondary metabolites modulating detoxification genes expression for natural red palm weevil pesticide development. Molecules 22, 169. 10.3390/molecules22010169 28117698 PMC6155707

[B4] Almasri H. Liberti J. Brunet J. L. Engel P. Belzunces L. P. (2022). Mild chronic exposure to pesticides alters physiological markers of honey bee health without perturbing the core gut microbiota. Sci. Rep. 12, 4281. 10.1038/s41598-022-08009-2 35277551 PMC8917129

[B5] Baron G. L. Jansen V. A. A. Brown M. J. F. Raine N. E. (2017). Pesticide reduces bumblebee colony initiation and increases probability of population extinction. Nat. Ecol. Evol. 1, 1308–1316. 10.1038/s41559-017-0260-1 29046553 PMC6485633

[B6] Berenbaum M. R. Johnson R. M. (2015). Xenobiotic detoxification pathways in honey bees. Curr. Opin. Insect Sci. 10, 51–58. 10.1016/j.cois.2015.03.005 29588014

[B7] Besard L. Mommaerts V. Abdu-Alla G. Smagghe G. (2011). Lethal and sublethal side-effect assessment supports a more benign profile of spinetoram compared with spinosad in the bumblebee *Bombus terrestris* . Pest Manag. Sci. 67, 541–547. 10.1002/ps.2093 21472971

[B8] Betz A. Andrew N. R. (2020). Influence of non-lethal doses of natural insecticides spinetoram and azadirachtin on *Helicoverpa punctigera*(Native Budworm, Lepidoptera: Noctuidae) under laboratory conditions. Front. Physiol. 11, 1089. 10.3389/fphys.2020.01089 32982799 PMC7485216

[B9] Cai H. L. Yang L. Zuo Z. P. Liao W. Y. Yang Z. X. (2021). Resistance status of *Myzus persicae* to pesticide and its relationship with enzymes. Agron. J. 113, 806–819. 10.1002/agj2.20490

[B10] Canche-Colli C. Estrella-Maldonado H. Medina-Medina L. A. Moo-Valle H. Calvo-Irabien L. M. Chan-Vivas E. (2021). Effect of yeast and essential oil-enriched diets on critical determinants of health and immune function in Africanized *Apis mellifera* . PeerJ 9, e12164. 10.7717/peerj.12164 34721958 PMC8522645

[B11] Chmiel J. A. Daisley B. A. Pitek A. P. Thompson G. J. Reid G. (2020). Understanding the effects of sublethal pesticide exposure on honey bees: A role for probiotics as mediators of environmental stress. Front. Ecol. Evol. 8, 22. 10.3389/fevo.2020.00022

[B12] Christen V. Kunz P. Y. Fent K. (2018). Endocrine disruption and chronic effects of plant protection products in bees: Can we better protect our pollinators? Environ. Pollut. 243, 1588–1601. 10.1016/j.envpol.2018.09.117 30296754

[B13] Cui P. Kong K. Yao Y. Huang Z. D. Shi S. P. Liu P. (2022). Community composition, bacterial symbionts, antibacterial and antioxidant activities of honeybee-associated fungi. BMC Microbiol. 22, 168. 10.1186/s12866-022-02580-4 35761187 PMC9235140

[B14] Daborn P. J. Yen J. L. Bogwitz M. R. Le Goff G. Feil E. Jeffers S. (2002). A single P450 allele associated with insecticide resistance in *Drosophila* . Science 297, 2253–2256. 10.1126/science.1074170 12351787

[B15] Dai P. L. Wang Q. Sun J. H. Liu F. Wang X. Wu Y. Y. (2010). Effects of sublethal concentrations of bifenthrin and deltamethrin on fecundity, growth, and development of the honeybee *Apis mellifera ligustica* . Environl Toxicol. Chem. 29, 644–649. 10.1002/etc.67 20821489

[B16] Demares F. J. Schmehl D. Bloomquist J. R. Cabrera A. R. Huang Z. Y. Lau P. (2022). Honey bee (*Apis mellifera*) exposure to pesticide residues in nectar and pollen in urban and suburban environments from four regions of the United States. Environl Toxicol. Chem. 41, 991–1003. 10.1002/etc.5298 35262221

[B17] Di Noi A. Casini S. Campani T. Cai G. M. Caliani I. (2021). Review on sublethal effects of environmental contaminants in honey bees (*Apis mellifera*), knowledge gaps and future perspectives. Int. J. Env. Res. Pub He 18, 1863. 10.3390/ijerph18041863 PMC7918799 33672936

[B18] Disayathanoowat T. Li H. Supapimon N. Suwannarach N. Lumyong S. Chantawannakul P. (2020). Different dynamics of bacterial and fungal communities in hive-stored bee bread and their possible roles: A case study from two commercial honey bees in China. Microorganisms 8, 264. 10.3390/microorganisms8020264 32075309 PMC7074699

[B19] Dong Z. X. Chen Y. F. Li H. Y. Tang Q. H. Guo J. (2021). The succession of the gut microbiota in insects: A dynamic alteration of the gut microbiota during the whole life cycle of honey bees (*Apis cerana*). Front. Microbiol. 12, 513962. 10.3389/fmicb.2021.513962 33935980 PMC8079811

[B20] Farder-Gomes C. F. Fernandes K. M. Bernardes R. C. Bastos D. S. S. de Oliveira L. L. M. Ferreira G. (2021). Harmful effects of fipronil exposure on the behavior and brain of the stingless bee *Partamona helleri* Friese (Hymenoptera: Meliponini). Sci. Total Environ. 794, 148678. 10.1016/j.scitotenv.2021.148678 34225147

[B21] Farooqi M. A. Mansoor ul H. Akhtar S. Arshad M. Aslam M. N. Rafay M. (2017). Detection of insecticide residues in honey of *Apis dorsata* F. from southern Punjab, Pakistan. Pak J. Zool. 49, 1761–1766. 10.17582/journal.pjz/2017.49.5.1761.1766

[B22] Gierer F. Vaughan S. Slater M. Thompson H. M. Elmore J. S. Girling R. D. (2019). A review of the factors that influence pesticide residues in pollen and nectar: Future research requirements for optimising the estimation of pollinator exposure. Environ. Pollut. 249, 236–247. 10.1016/j.envpol.2019.03.025 30893636

[B23] Gill R. J. Raine N. E. (2014). Chronic impairment of bumblebee natural foraging behaviour induced by sublethal pesticide exposure. Funct. Ecol. 28, 1459–1471. 10.1111/1365-2435.12292

[B24] Gill R. J. Ramos-Rodriguez O. Raine N. E. (2012). Combined pesticide exposure severely affects individual- and colony-level traits in bees. Nature 491, 105–108. 10.1038/nature11585 23086150 PMC3495159

[B25] Green J. M. (2016). The rise and future of glyphosate and glyphosate-resistant crops. Pest Manag. Sci. 74, 1035–1039. 10.1002/ps.4462 27758090

[B26] Hammer T. J. Le E. Martin A. N. Moran N. A. (2021). The gut microbiota of bumblebees. Insect Soc. 68, 287–301. 10.1007/s00040-021-00837-1 PMC8956082 35342195

[B27] Herbert L. T. Vazquez D. E. Arenas A. Farina W. M. (2014). Effects of field-realistic doses of glyphosate on honeybee appetitive behaviour. J. Exp. Biol. 217, 3457–3464. 10.1242/jeb.109520 25063858

[B28] Hsu C. K. Wang D. Y. Wu M. C. (2021). A potential fungal probiotic *Aureobasidium melanogenum* CK-CsC for the Western honey bee, *Apis mellifera* . J. Fungi 7, 508. 10.3390/jof7070508 PMC8306588 34202244

[B29] Jabot C. Fieu M. Giroud B. Bulete A. Casabianca H. Vulliet E. (2015). Trace-level determination of pyrethroid, neonicotinoid and carboxamide pesticides in beeswax using dispersive solid-phase extraction followed by ultra-high-performance liquid chromatography-tandem mass spectrometry. Int J Environ Ch 95, 240–257. 10.1080/03067319.2015.1016011

[B30] Jiang D. Wu S. Tan M. T. Wang Q. Zheng L. Yan S. C. (2021). The high adaptability of *Hyphantria cunea* larvae to cinnamic acid involves in detoxification, antioxidation and gut microbiota response. Pestic. Biochem. Phys. 174, 104805. 10.1016/j.pestbp.2021.104805 33838706

[B31] Kilani-Morakchi S. Bezzar-Bendjazia R. Ferdenache M. Aribi N. (2017). Preimaginal exposure to azadirachtin affects food selection and digestive enzymes in adults of *Drosophila melanogaster* (Diptera: Drosophilidae). Pestic. Biochem. Physiol. 140, 58–64. 10.1016/j.pestbp.2017.06.004 28755695

[B32] Koch H. Schmid-Hempel P. (2011). Bacterial communities in central European bumblebees: Low diversity and high specificity. Microb. Ecol. 62, 121–133. 10.1007/s00248-011-9854-3 21556885

[B33] Kokuvo N. Toquenaga Y. Goka K. (2008). Estimating colony number of *Bombus terrestris* (hymenoptera, apidae) queens foraging in biratori, hokkaido, Japan. Appl. Entomol. Zool. 43, 19–23. 10.1303/aez.2008.19

[B34] Li L. Solvi C. Zhang F. Qi Z. Y. Chittka L. Zhao W. (2021). Gut microbiome drives individual memory variation in bumblebees. Nat. Commun. 12, 6588. 10.1038/s41467-021-26833-4 34824201 PMC8616916

[B35] Li Z. G. Li M. He J. F. Zhao X. M. Chaimanee V. Huang W. F. (2017). Differential physiological effects of neonicotinoid insecticides on honey bees: A comparison between *Apis mellifera* and *Apis cerana* . Pestic. Biochem. Physiol. 140, 1–8. 10.1016/j.pestbp.2017.06.010 28755688

[B36] Liu Y. J. Qiao N. H. Diao Q. Y. Jing Z. Vukanti R. Dai P. L. (2020). Thiacloprid exposure perturbs the gut microbiota and reduces the survival status in honeybees. J. Hazard Mater 389, 121818. 10.1016/j.jhazmat.2019.121818 31818660

[B37] Motta E. V. S. Mak M. De Jong T. K. Powell J. E. O'Donnell A. Suhr K. J. (2020). Oral or topical exposure to glyphosate in herbicide formulation impacts the gut microbiota and survival rates of honey bees. Appl. Environ. Microb. 86, e01150–e01120. 10.1128/AEM.01150-20 PMC7480383 32651208

[B38] Motta E. V. S. Raymann K. Moran N. A. (2018). Glyphosate perturbs the gut microbiota of honey bees. Proc. Natl. Acad. Sci. U. S. A. 115, 10305–10310. 10.1073/pnas.1803880115 30249635 PMC6187125

[B68] Murawska A. Migdał P. Roman A. (2021). Effects of plant protection products on biochemical markers in honey bees. Agriculture-Basel. 11, 648.

[B39] Nieto A. Roberts S. P. M. Kemp J. Rasmont P. Kuhlmann M. Garcı´a Criado M. (2014). European red list of bees. Luxembourg: Publication Office of the European Union.

[B40] Odemer R. Alkassab A. T. Bischoff G. Frommberger M. Wernecke A. Wirtz I. P. (2020). Chronic high glyphosate exposure delays individual worker bee (*Apis mellifera* L.) development under field conditions. Insects 11, 664. 10.3390/insects11100664 32992639 PMC7600025

[B41] Perez-Cobo I. Fernandez-Alba A. R. Hernando D. (2022). First national survey of residues of active substances in honeybee apiaries across Spain between 2012 and 2016. Sci. Total Environ. 838, 155614. 10.1016/j.scitotenv.2022.155614 35504369

[B42] Piovesan B. Padilha A. C. Morais M. C. Botton M. Grutzmacher A. D. Zotti M. J. (2020). Effects of insecticides used in strawberries on stingless bees *Melipona quadrifasciata* and *Tetragonisca fiebrigi*(Hymenoptera: Apidae). Environ. Sci. Pollut. R. 27, 42472–42480. 10.1007/s11356-020-10191-7 32705562

[B43] Rutkowski D. Litsey E. Maalouf I. Vannette R. L. (2022). Bee-associated fungi mediate effects of fungicides on bumble bees. Ecol. Entomol. 47, 411–422. 10.1111/een.13126

[B44] Schonbrunn E. Eschenburg S. Shuttleworth W. A. Schloss J. V. Amrhein N. Evans J. N. S. (2001). Interaction of the herbicide glyphosate with its target enzyme 5-enolpyruvylshikimate 3-phosphate synthase in atomic detail. Proc. Natl. Acad. Sci. USA. 98, 1376–1380. 10.1073/pnas.98.4.1376 11171958 PMC29264

[B45] Smith D. B. Arce A. N. Rodrigues A. R. Bischoff P. H. Burris D. Ahmed F. (2020). Insecticide exposure during brood or early-adult development reduces brain growth and impairs adult learning in bumblebees. P Roy. Soc. B-Biol Sci. 287, 20192442. 10.1098/rspb.2019.2442 PMC7126076 32126960

[B46] Song Y. Shi J. Xiong Z. Shentu X. Yu X. (2021). Three antimicrobials alter gut microbial communities and causing different mortality of Brown planthopper, *Nilaparvata lugens* Stal. Pestic. Biochem. Physiol. 174, 104806. 10.1016/j.pestbp.2021.104806 33838707

[B47] Soroye P. Newbold T. Kerr J. (2020). Climate change contributes to widespread declines among bumble bees across continents. Science 367, 685–688. 10.1126/science.aax8591 32029628

[B48] Straw E. A. Carpentier E. N. Brown M. J. F. (2021). Roundup causes high levels of mortality following contact exposure in bumble bees. J. Appl. Ecol. 58, 1167–1176. 10.1111/1365-2664.13867

[B49] Takeuchi T. Takahashi M. Nishimoto M. Kiyoshi T. Tsuchida K. Nomura T. (2018). Genetic structure of the bumble bee *Bombus hypocrita* sapporoensis, a potential domestic pollinator for crops in Japan. J. Apic. Res. 57, 203–212. 10.1080/00218839.2017.1412879

[B50] Tang Q. H. Miao C. H. Chen Y. F. Dong Z. X. Cao Z. Liao S. Q. (2021). The composition of bacteria in gut and beebread of stingless bees (Apidae: Meliponini) from tropics Yunnan, China. Ant. Leeuw Int. J. G. 114, 1293–1305. 10.1007/s10482-021-01602-x 34110551

[B51] Tasman K. Rands S. A. Hodge J. J. L. (2020). The neonicotinoid insecticide imidacloprid disrupts bumblebee foraging rhythms and sleep. iScience 23, 101827. 10.1016/j.isci.2020.101827 33305183 PMC7710657

[B52] Thompson H. M. Levine S. L. Doering J. Norman S. Manson P. Sutton P. (2014). Evaluating exposure and potential effects on honeybee brood (*Apis mellifera*) development using glyphosate as an example. Integr. Environ. Asses 10, 463–470. 10.1002/ieam.1529 PMC4285224 24616275

[B53] Trestrail C. Walpitagama M. Miranda A. Nugegoda D. Shimeta J. (2021). Microplastics alter digestive enzyme activities in the marine bivalve, *Mytilus galloprovincialis* . Sci. Total Environ. 779, 146418. 10.1016/j.scitotenv.2021.146418 33744572

[B54] Tsvetkov N. MacPhail V. J. Colla S. R. Zayed A. (2021). Conservation genomics reveals pesticide and pathogen exposure in the declining bumble bee *Bombus terricola* . Mol. Ecol. 30, 4220–4230. 10.1111/mec.16049 34181797 PMC8457087

[B55] Vazquez D. E. Ilina N. Pagano E. A. Zavala J. A. Farina W. M. (2018). Glyphosate affects the larval development of honey bees depending on the susceptibility of colonies. PLoS One 13, e0205074. 10.1371/journal.pone.0205074 30300390 PMC6177133

[B56] Vyjayanthi N. Subramanyam M. V. V. (2002). Effect of fenvalerate-20EC on sericigenous insects II. Digestive enzymes in the nutritive physiology of silkworm, *Bombyx mori* L. . Ecotox Environ. Safe. 53, 212–220. 10.1006/eesa.2002.2229 12568456

[B57] Wang W. L. Gao C. L. Ren L. L. Luo Y. Q. (2019a). The effect of longwave ultraviolet light radiation on *Dendrolimus tabulaeformis* antioxidant and detoxifying enzymes. Insects 11, 1. 10.3390/insects11010001 31861292 PMC7022865

[B58] Wang Y. F. Zhang W. X. Shi T. F. Xu S. Y. Lu B. Z. Qin H. W. (2019b). Synergistic toxicity and physiological impact of thiamethoxam alone or in binary mixtures with three commonly used insecticides on honeybee. Apidologie 51, 395–405. 10.1007/s13592-019-00726-4

[B59] Weidenmuller A. Meltzer A. Neupert S. Schwarz A. Kleineidam C., 2022. Glyphosate impairs collective thermoregulation in bumblebees. Science. 376, 1122–1126. 10.1126/science.abf7482 35653462

[B60] Wen X. L. Ma C. S. Sun M. H. Wang Y. Xue X. F. Chen J. (2021). Pesticide residues in the pollen and nectar of oilseed rape (*Brassica napus* L.) and their potential risks to honey bees. Sci. Total Environ. 786, 147443. 10.1016/j.scitotenv.2021.147443 33965824

[B61] Xiao J. J. He Q. B. Liu Q. Q. Wang Z. Y. Yin F. Chai Y. H. (2022). Analysis of honey bee exposure to multiple pesticide residues in the hive environment. Sci. Total Environ. 805, 150292. 10.1016/j.scitotenv.2021.150292 34536857

[B62] Yang Y. Ma S. L. Yan Z. X. Liu F. Diao Q. Y. Dai P. L. (2019). Effects of three common pesticides on survival, food consumption and midgut bacterial communities of adult workers *Apis cerana* and *Apis mellifera* . Environ. Pollut. 249, 860–867. 10.1016/j.envpol.2019.03.077 30954834

[B63] Yun J. H. Jung M. J. Kim P. S. Bae J. W. (2018). Social status shapes the bacterial and fungal gut communities of the honey bee. Sci. Rep. 8, 2019. 10.1038/s41598-018-19860-7 29386588 PMC5792453

[B64] Zhang Q. L. Li H. W. Wu W. Zhang M. Guo J. Deng X. Y. (2019). The response of microbiota community to streptococcus agalactiae infection in zebrafish intestine. Front. Microbiol. 10, 2848. 10.3389/fmicb.2019.02848 31866993 PMC6908962

[B65] Zhang R. Y. Bai X. H. Shao J. H. Chen A. W. Wu H. Y. Luo S. (2020). Effects of zero-valent iron nanoparticles and quinclorac coexposure on the growth and antioxidant system of rice (*Oryza sativa* L.). Ecotoxicol. Environ. Saf. 203, 111054. 10.1016/j.ecoenv.2020.111054 32888616

[B66] Zhu L. Z. Qi S. Z. Xue X. F. Niu X. Y. Wu L. M. (2020a). Nitenpyram disturbs gut microbiota and influences metabolic homeostasis and immunity in honey bee (*Apis mellifera* L.). Environ. Pollut. 258, 113671. 10.1016/j.envpol.2019.113671 31855676

[B67] Zhu Y. C. Caren J. Reddy G. V. P. Li W. H. Yao J. X. (2020b). Effect of age on insecticide susceptibility and enzymatic activities of three detoxification enzymes and one invertase in honey bee workers (*Apis mellifera*). Comp. Biochem. Phys. C 238, 108844. 10.1016/j.cbpc.2020.108844 32777468

