# Supplementary material for: Effects of spinetoram and glyphosate on physiological biomarkers and gut microbes in Bombus terrestris
Source: Front Physiol. 2023 Jan 9;13:1054742. doi: 10.3389/fphys.2022.1054742 (PMC9868390; doi:10.3389/fphys.2022.1054742)
Supplement: Supplementary file 1 [file Table3.doc]

**Table S3** Major fungi in the gut of bumblebees at the phylum and genus level

| Classification | | Control group | Glyphosate group | *P* - value |
| --- | --- | --- | --- | --- |
| Phylum | Ascomycota | 98.84 ± 0.54 | 72.34 ± 5.09 | <0.001 |
|  | Basidiomycota | 0.46 ± 0.14 | 1.79 ± 0.04 | <0.001 |
| Genus | *Zygosaccharomyces* | 96.83 ± 0.74 | 60.14 ± 5.32 | <0.001 |
|  | *Cladosporium* | 0.95 ± 0.43 | 5.05 ± 0.34 | <0.001 |
|  | *Botrytis* | 0.04 ± 0.04 | 0.90 ± 0.77 | 0.128 |
|  | *Naganishia* | 0.01 ± 0.01 | 0.62 ± 1.03 | 0.361 |
|  | *Kodamaea* | 0.36 ± 0.57 | 0.04 ± 0.03 | 0.375 |

The numeric values are expressed as the mean ± standard deviation (SD). Significant differences between the two groups were tested by the Student‘s t-test (n=3).
