# Supplementary figures and images for: Effects of spinetoram and glyphosate on physiological biomarkers and gut microbes in Bombus terrestris
Source: Front Physiol. 2023 Jan 9;13:1054742. doi: 10.3389/fphys.2022.1054742 (PMC9868390; doi:10.3389/fphys.2022.1054742)

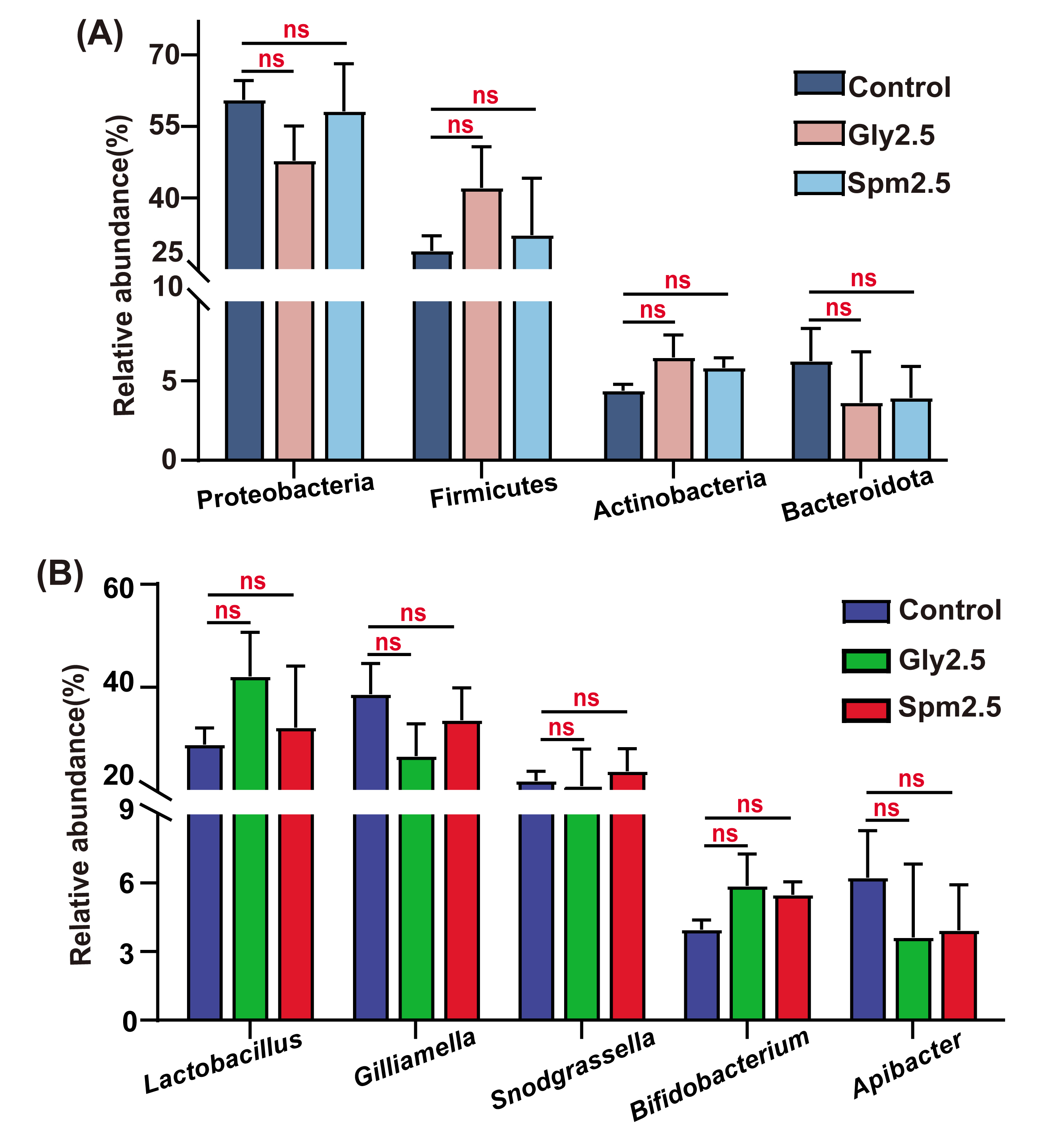

Supplement: Supplementary file 2 [file Image1.JPEG]
