# Supplementary material for: Effects of spinetoram and glyphosate on physiological biomarkers and gut microbes in Bombus terrestris
Source: Front Physiol. 2023 Jan 9;13:1054742. doi: 10.3389/fphys.2022.1054742 (PMC9868390; doi:10.3389/fphys.2022.1054742)
Supplement: Supplementary file 3 [file Table1.doc]

**Table S1** Bacterial alpha diversity of each bumblebee gut sample

| Sample | coverage | Alpha diversity | |
| --- | --- | --- | --- |
|  |  | chao | simpson |
| Control 1 | 0.99997 | 21 | 0.18997 |
| Control 2 | 0.99997 | 19 | 0.23852 |
| Control 3 | 0.99992 | 25 | 0.20493 |
| Glyphosate 1 | 0.99983 | 39 | 0.20315 |
| Glyphosate 2 | 0.99989 | 28 | 0.23070 |
| Glyphosate 3 | 0.99994 | 23 | 0.22807 |
| Spinetoram 1 | 1 | 20 | 0.21707 |
| Spinetoram 2 | 0.99994 | 23 | 0.16563 |
| Spinetoram 3 | 0.99994 | 23 | 0.14142 |
