# Supplementary material for: Effects of spinetoram and glyphosate on physiological biomarkers and gut microbes in Bombus terrestris
Source: Front Physiol. 2023 Jan 9;13:1054742. doi: 10.3389/fphys.2022.1054742 (PMC9868390; doi:10.3389/fphys.2022.1054742)
Supplement: Supplementary file 4 [file Table2.doc]

**Table S2** Fungal alpha diversity of each bumblebee gut sample

| Sample | coverage | Alpha diversity | |
| --- | --- | --- | --- |
|  |  | chao | simpson |
| Control 1 | 1 | 54 | 0.95438 |
| Control 2 | 0.99998 | 50 | 0.92987 |
| Control 3 | 0.99994 | 55 | 0.92919 |
| Glyphosate 1 | 0.99986 | 75 | 0.33601 |
| Glyphosate 2 | 0.99992 | 59 | 0.45028 |
| Glyphosate 3 | 0.99998 | 46 | 0.43107 |
